# 2-Hy­droxy­ethyl 4-hy­droxy­benzoate

**DOI:** 10.1107/S1600536810054516

**Published:** 2011-01-22

**Authors:** M. Jeyalaxmi, G. Jagadeesan, J. Arulmoli, D. Roop Singh, S. Aravindhan

**Affiliations:** a Department of Physics, Presidency College, Chennai 600 005, India; b Department of Chemistry, Presidency College, Chennai 600 005, India

## Abstract

In the title compound, C_9_H_10_O_4_, the dihedral angle between the benzene ring and the –CO_2_ unit is 11.93 (8)° and the conformation of the 2-hy­droxy­ethyl side chain is *gauche* [O—C—C—O = −71.91 (17)°]. In the crystal, mol­ecules are linked by O—H⋯O and C—H⋯O hydrogen bonds.

## Related literature

For background to the properties of esters of 4-hy­droxy­benzoic acid, see: Kadokawa *et al.* (2002 ▶).
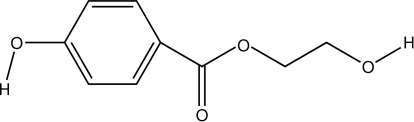


## Experimental

### 

#### Crystal data

C_9_H_10_O_4_
*M* *_r_* = 182.17
Triclinic, 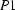

*a* = 4.4235 (10) Å
*b* = 5.6850 (17) Å
*c* = 8.7050 (17) Å
α = 80.819 (13)°
β = 79.943 (14)°
γ = 81.804 (14)°
*V* = 211.30 (9) Å^3^
*Z* = 1
Mo *K*α radiation
μ = 0.11 mm^−1^
*T* = 293 K
0.20 × 0.20 × 0.20 mm

#### Data collection

Bruker SMART APEXII CCD diffractometer
Absorption correction: multi-scan (*SADABS*; Bruker, 2008 ▶) *T*_min_ = 0.978, *T*_max_ = 0.982
3761 measured reflections
1767 independent reflections
1609 reflections with *I* > 2σ(*I*)
*R*_int_ = 0.018

#### Refinement

*R*[*F* ^2^ > 2σ(*F* ^2^)] = 0.031
*wR*(*F* ^2^) = 0.088
*S* = 1.06
1767 reflections
126 parameters
3 restraints
H atoms treated by a mixture of independent and constrained refinement
Δρ_max_ = 0.21 e Å^−3^
Δρ_min_ = −0.14 e Å^−3^

### 

Data collection: *APEX2* (Bruker, 2008 ▶); cell refinement: *SAINT* (Bruker, 2008 ▶); data reduction: *SAINT*; program(s) used to solve structure: *SHELXS97* (Sheldrick, 2008 ▶); program(s) used to refine structure: *SHELXL97* (Sheldrick, 2008 ▶); molecular graphics: *ORTEP-3* (Farrugia, 1997 ▶); software used to prepare material for publication: *SHELXL97* and *PLATON* (Spek, 2009 ▶).

## Supplementary Material

Crystal structure: contains datablocks I, global. DOI: 10.1107/S1600536810054516/hb5779sup1.cif

Structure factors: contains datablocks I. DOI: 10.1107/S1600536810054516/hb5779Isup2.hkl

Additional supplementary materials:  crystallographic information; 3D view; checkCIF report

## Figures and Tables

**Table 1:** Hydrogen-bond geometry (Å, °)

*D*—H⋯*A*	*D*—H	H⋯*A*	*D*⋯*A*	*D*—H⋯*A*
O1—H1*A*⋯O4^i^	0.86 (3)	1.87 (3)	2.7204 (19)	169 (2)
O4—H4*A*⋯O2^ii^	0.75 (3)	2.15 (3)	2.8970 (18)	170 (3)
C9—H9*A*⋯O2^iii^	0.97	2.51	3.322 (2)	141

Symmetry codes: (i) 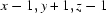; (ii) 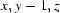; (iii) 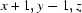.

**Fig. 1.**
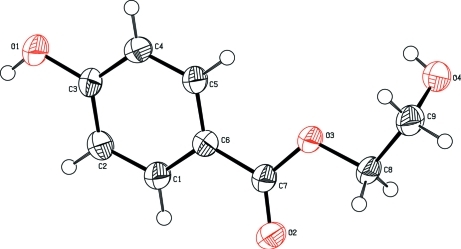
The molecular structure of (I) showing 30% probability displacement ellipsoids.

**Fig. 2.**
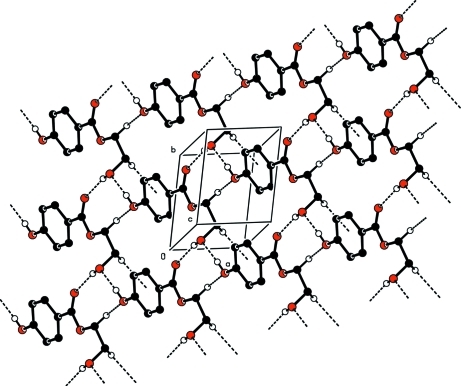
Crystal packing diagram. H atoms not involved in hydrogen bonding (dashed lines) have been omitted for clarity.

## supplementary crystallographic information

### Comment

The *ORTEP* diagram of the title compound, (I), shown in Fig.1 indicates that the aromatic ring is in a plane and the ester group attached to it maintains near planarity with it which is defined by the torsion angles C5—C6—C7—O2 (166.91°), C5—C6—C7—O3 (-12.81°).

Though the C6—C7 is a single bond (1.468 Å) and the possibility of free rotation is high at that connectivity, the planarity exerted by the ester group may be purely because of crystal packing.

The torsion angle O3—C8—C9—O4 is -71.09° which makes the ethyl hydroxy O4 to assume the *syn*-clinal conformation with respect to the carboxy O3. Such a conformation instead of anti conformation may be due to crystal packing of the molecules which makes them compactly stacked to one another.

The crystal packing (Fig.2) shows the presence of inter-molecular hydrogen bonding. The phenolic oxygen (O1) forms a strong intermolecular hydrogen bond (O1—H1A···O4) with the D···A distance of 2.720 Å and the D—H···A angle of 169°. The ethanolic O4 donates the hydrogen to symmetrically related carbonyl O2 to form intermolecular hydrogen bond (O4—H4A···O2) with the D···A distance of 2.897 Å and the D—H···A angle of 170°. The carbon (C9) atom forms a weak intermolecular hydrogen bond (C9—H9A···O2) with the D···A distance of 3.392 Å and the D—H···A angle is 140.8°. All these three hydrogen bonds are exisiting between a given molecule and three different symmetry related molecules (*x* - 1, +*y* + 1, +*z* - 1 *x*, +*y* - 1, +*z* and *x* + 1, +*y* - 1, +*z* respectively). This multiple hydrogen bonding network makes the well defined crystal packing.

### Experimental

An ethanolic solution of 3-methyl-1-phenyl-4-acetylpyrazolin-5-ol (0.432 g, 2 mmol) and 2-aminoethanol (0.122 g, 2mmoL) were taken in a round bottom flask and refluxed for 4 h. The solid product was filtered and washed with cold ethanol. The product obtained was pure by TLC and NMR spectroscopy. However, the product was further purified by re-crystallization from ethanol and dried under vacuum. The compound was crystallized by slow evaporation technique using methanol as solvent at room temperature to yield colourless blocks of (I).

### Refinement

Anomalous dispersion was negliglble and the absolute sturcture of (I) could not be determined in the present analysis.

### Crystal data

**Table:**

C_9_H_10_O_4_	*Z* = 1
*M**_r_* = 182.17	*F*(000) = 96
Triclinic, *P*1	*D*_x_ = 1.432 Mg m^−^^3^
Hall symbol: P 1	Mo *K*α radiation, λ = 0.71073 Å
*a* = 4.4235 (10) Å	Cell parameters from 1767 reflections
*b* = 5.6850 (17) Å	θ = 2.4–28.3°
*c* = 8.7050 (17) Å	µ = 0.11 mm^−^^1^
α = 80.819 (13)°	*T* = 293 K
β = 79.943 (14)°	Block, colourless
γ = 81.804 (14)°	0.20 × 0.20 × 0.20 mm
*V* = 211.30 (9) Å^3^	

### Data collection

**Table:**

Bruker SMART APEXII CCD diffractometer	1767 independent reflections
Radiation source: fine-focus sealed tube	1609 reflections with *I* > 2σ(*I*)
graphite	*R*_int_ = 0.018
ω scans	θ_max_ = 28.3°, θ_min_ = 2.4°
Absorption correction: multi-scan (*SADABS*; Bruker, 2008)	*h* = −5→5
*T*_min_ = 0.978, *T*_max_ = 0.982	*k* = −7→7
3761 measured reflections	*l* = −11→10

### Refinement

**Table:**

Refinement on *F*^2^	Primary atom site location: structure-invariant direct methods
Least-squares matrix: full	Secondary atom site location: difference Fourier map
*R*[*F*^2^ > 2σ(*F*^2^)] = 0.031	Hydrogen site location: inferred from neighbouring sites
*wR*(*F*^2^) = 0.088	H atoms treated by a mixture of independent and constrained refinement
*S* = 1.06	*w* = 1/[σ^2^(*F*_o_^2^) + (0.0532*P*)^2^ + 0.0112*P*] where *P* = (*F*_o_^2^ + 2*F*_c_^2^)/3
1767 reflections	(Δ/σ)_max_ < 0.001
126 parameters	Δρ_max_ = 0.21 e Å^−^^3^
3 restraints	Δρ_min_ = −0.14 e Å^−^^3^

### Special details

**Table:**

Geometry. All e.s.d.'s (except the e.s.d. in the dihedral angle between two l.s. planes) are estimated using the full covariance matrix. The cell e.s.d.'s are taken into account individually in the estimation of e.s.d.'s in distances, angles and torsion angles; correlations between e.s.d.'s in cell parameters are only used when they are defined by crystal symmetry. An approximate (isotropic) treatment of cell e.s.d.'s is used for estimating e.s.d.'s involving l.s. planes.
Refinement. Refinement of *F*^2^ against ALL reflections. The weighted *R*-factor *wR* and goodness of fit *S* are based on *F*^2^, conventional *R*-factors *R* are based on *F*, with *F* set to zero for negative *F*^2^. The threshold expression of *F*^2^ > σ(*F*^2^) is used only for calculating *R*-factors(gt) *etc*. and is not relevant to the choice of reflections for refinement. *R*-factors based on *F*^2^ are statistically about twice as large as those based on *F*, and *R*- factors based on ALL data will be even larger.

### Fractional atomic coordinates and isotropic or equivalent isotropic displacement parameters (Å^2^)

**Table:**

	*x*	*y*	*z*	*U*_iso_*/*U*_eq_	
O1	0.5796 (3)	0.5763 (2)	0.61019 (14)	0.0532 (3)	
O2	1.0292 (3)	0.7852 (2)	1.22266 (15)	0.0481 (3)	
O3	1.1612 (2)	0.38911 (18)	1.22823 (13)	0.0413 (3)	
O4	1.2540 (3)	−0.0314 (2)	1.46819 (18)	0.0527 (3)	
C1	0.7231 (4)	0.7899 (3)	0.96121 (18)	0.0389 (4)	
H1	0.6776	0.9251	1.0126	0.047*	
C2	0.6084 (4)	0.7891 (3)	0.82391 (19)	0.0416 (4)	
H2	0.4835	0.9223	0.7836	0.050*	
C3	0.6800 (3)	0.5888 (2)	0.74610 (16)	0.0383 (4)	
C4	0.8620 (4)	0.3879 (3)	0.80814 (19)	0.0441 (4)	
H4	0.9092	0.2533	0.7562	0.053*	
C5	0.9712 (4)	0.3888 (3)	0.94576 (17)	0.0406 (4)	
H5	1.0900	0.2534	0.9878	0.049*	
C6	0.9064 (3)	0.5905 (2)	1.02359 (17)	0.0346 (3)	
C7	1.0346 (3)	0.6029 (3)	1.16625 (17)	0.0342 (3)	
C8	1.3041 (4)	0.3878 (3)	1.36534 (18)	0.0385 (3)	
H8A	1.1488	0.4292	1.4532	0.046*	
H8B	1.4522	0.5040	1.3436	0.046*	
C9	1.4636 (4)	0.1408 (3)	1.4041 (2)	0.0447 (4)	
H9A	1.5896	0.0912	1.3092	0.054*	
H9B	1.6004	0.1443	1.4793	0.054*	
H1A	0.471 (6)	0.706 (5)	0.577 (3)	0.058 (6)*	
H4A	1.190 (5)	−0.062 (4)	1.401 (3)	0.056 (7)*	

### Atomic displacement parameters (Å^2^)

**Table:**

	*U*^11^	*U*^22^	*U*^33^	*U*^12^	*U*^13^	*U*^23^
O1	0.0782 (9)	0.0440 (7)	0.0442 (7)	0.0023 (6)	−0.0365 (6)	−0.0051 (5)
O2	0.0672 (8)	0.0375 (6)	0.0458 (6)	0.0033 (5)	−0.0264 (5)	−0.0130 (4)
O3	0.0587 (7)	0.0341 (5)	0.0368 (6)	−0.0018 (4)	−0.0256 (5)	−0.0055 (4)
O4	0.0742 (8)	0.0404 (6)	0.0511 (8)	−0.0043 (5)	−0.0365 (6)	−0.0024 (5)
C1	0.0488 (9)	0.0333 (8)	0.0360 (8)	0.0007 (6)	−0.0148 (6)	−0.0057 (6)
C2	0.0505 (9)	0.0336 (7)	0.0414 (8)	0.0019 (6)	−0.0188 (7)	−0.0010 (6)
C3	0.0498 (9)	0.0376 (8)	0.0306 (8)	−0.0064 (6)	−0.0173 (7)	−0.0003 (6)
C4	0.0631 (10)	0.0324 (7)	0.0415 (9)	−0.0008 (7)	−0.0219 (7)	−0.0081 (6)
C5	0.0553 (9)	0.0320 (8)	0.0375 (9)	0.0018 (6)	−0.0223 (7)	−0.0034 (6)
C6	0.0410 (8)	0.0324 (7)	0.0323 (8)	−0.0061 (6)	−0.0106 (6)	−0.0035 (6)
C7	0.0381 (8)	0.0357 (8)	0.0305 (7)	−0.0026 (6)	−0.0116 (6)	−0.0041 (6)
C8	0.0488 (9)	0.0385 (8)	0.0330 (7)	−0.0008 (6)	−0.0209 (6)	−0.0075 (6)
C9	0.0508 (9)	0.0426 (8)	0.0443 (9)	0.0060 (6)	−0.0247 (7)	−0.0080 (6)

### Geometric parameters (Å, °)

**Table:**

O1—C3	1.3494 (19)	C3—C4	1.392 (2)
O1—H1A	0.86 (3)	C4—C5	1.369 (2)
O2—C7	1.211 (2)	C4—H4	0.9300
O3—C7	1.3357 (17)	C5—C6	1.394 (2)
O3—C8	1.4436 (18)	C5—H5	0.9300
O4—C9	1.426 (2)	C6—C7	1.468 (2)
O4—H4A	0.75 (3)	C8—C9	1.494 (2)
C1—C2	1.379 (2)	C8—H8A	0.9700
C1—C6	1.390 (2)	C8—H8B	0.9700
C1—H1	0.9300	C9—H9A	0.9700
C2—C3	1.387 (2)	C9—H9B	0.9700
C2—H2	0.9300		
			
C3—O1—H1A	112.3 (16)	C1—C6—C5	119.03 (13)
C7—O3—C8	115.86 (12)	C1—C6—C7	118.75 (13)
C9—O4—H4A	107.0 (19)	C5—C6—C7	122.18 (12)
C2—C1—C6	120.59 (14)	O2—C7—O3	123.00 (14)
C2—C1—H1	119.7	O2—C7—C6	124.52 (13)
C6—C1—H1	119.7	O3—C7—C6	112.48 (12)
C1—C2—C3	119.71 (14)	O3—C8—C9	107.48 (12)
C1—C2—H2	120.1	O3—C8—H8A	110.2
C3—C2—H2	120.1	C9—C8—H8A	110.2
O1—C3—C2	123.02 (14)	O3—C8—H8B	110.2
O1—C3—C4	116.90 (13)	C9—C8—H8B	110.2
C2—C3—C4	120.08 (13)	H8A—C8—H8B	108.5
C5—C4—C3	119.84 (14)	O4—C9—C8	113.01 (14)
C5—C4—H4	120.1	O4—C9—H9A	109.0
C3—C4—H4	120.1	C8—C9—H9A	109.0
C4—C5—C6	120.74 (13)	O4—C9—H9B	109.0
C4—C5—H5	119.6	C8—C9—H9B	109.0
C6—C5—H5	119.6	H9A—C9—H9B	107.8
			
C6—C1—C2—C3	−0.9 (2)	C4—C5—C6—C7	−176.35 (15)
C1—C2—C3—O1	−179.07 (13)	C8—O3—C7—O2	−2.2 (2)
C1—C2—C3—C4	1.2 (2)	C8—O3—C7—C6	177.51 (12)
O1—C3—C4—C5	−179.98 (15)	C1—C6—C7—O2	−10.8 (2)
C2—C3—C4—C5	−0.3 (2)	C5—C6—C7—O2	166.91 (15)
C3—C4—C5—C6	−1.0 (3)	C1—C6—C7—O3	169.50 (13)
C2—C1—C6—C5	−0.4 (2)	C5—C6—C7—O3	−12.81 (18)
C2—C1—C6—C7	177.41 (14)	C7—O3—C8—C9	−173.21 (12)
C4—C5—C6—C1	1.3 (2)	O3—C8—C9—O4	−71.91 (17)

### Hydrogen-bond geometry (Å, °)

**Table:**

*D*—H···*A*	*D*—H	H···*A*	*D*···*A*	*D*—H···*A*
O1—H1A···O4^i^	0.86 (3)	1.87 (3)	2.7204 (19)	169 (2)
O4—H4A···O2^ii^	0.75 (3)	2.15 (3)	2.8970 (18)	170 (3)
C9—H9A···O2^iii^	0.97	2.51	3.322 (2)	141

Symmetry codes: (i) *x*−1, *y*+1, *z*−1; (ii) *x*, *y*−1, *z*; (iii) *x*+1, *y*−1, *z*.
